# Knowledge of companion animals’ practitioners on stem-cell based therapies in a clinical context: a questionnaire-based survey in Portugal

**DOI:** 10.1186/s12917-025-04872-z

**Published:** 2025-07-24

**Authors:** Rafael S. Lopes, João Requicha, Nuno Carolino, Eduardo Costa, Pedro Carvalho

**Affiliations:** 1 Department of Veterinary Sciences, University of Trás-os-Montes e Alto Douro (UTAD), Quinta de Prados, Vila Real, 5000-801 Portugal; 2 Vasco da Gama Research Center (CIVG), Vasco da Gama University School (EUVG), Campus Universitário de Lordemão, Av. José R. Sousa Fernandes, Coimbra, 3020-210 Portugal; 3 Animal and Veterinary Research Centre (CECAV) - Associate Laboratory for Animal and Veterinary Science (AL4AnimalS), UTAD, Vila Real, Portugal; 4 Estação Zootécnica Nacional - Polo de Investigação da Fonte Boa, Vale de Santarém, Santarém, 2005-048 Portugal; 5 Vetherapy– Research & Development in Biotechnology, Coimbra, Portugal; 6 Institute of Experimental Pathology, Faculty of Medicine, University of Coimbra, Azinhaga de Santa Comba, Coimbra, 3000-548 Portugal; 7 Chemistry Department, CQC-IMS, University of Coimbra, Rua Larga, Coimbra, 3004-535 Portugal

**Keywords:** Questionnaire-based survey, Knowledge, Stem cells, Cell therapies, Portugal

## Abstract

Stem cells therapies are a promising therapeutic option, and although not yet widely used in daily clinical practice in companion animals, they have shown great potential in the treatment of a variety of diseases. This study aims to investigate the individual profile of Portuguese veterinary practitioners, their knowledge about this type of therapy and their interest in its future use and application. This questionnaire-based descriptive study was carried out in Portugal from November 2021 to January 2022. A questionnaire-based survey was conducted online, using an electronic platform. The following topics were surveyed: (i) profile of veterinarians; (ii) knowledge about cell therapies; and (iii) interest in future use and application. After an internal validation, the survey was released online for three months in Portuguese veterinary social network groups. Data were collected and descriptive analysis and Chi-square test were performed. A total of 275 responses were obtained and analysed. Analysis of the results showed that the participating veterinarians know what mesenchymal stromal cells (MSCs) are (90.9%) and are generally aware of their therapeutic potential (94.5%). Regarding their use, 56.7% of the respondents do not know their application in veterinary pathologies. The remaining 43.3% indicated the situations in which they knew of its use. Orthopaedic and dental or oral diseases were the most frequently mentioned. Regarding the immunological effect of MSCs, there is a great lack of information. Data analysis showed that 72.4% of respondents were unaware of the possibility of an immunogenic response following their administration. However, 53.5% stated that they were aware of the positive immunomodulatory capacity. Most professionals who participated in this survey consider this topic relevant in veterinary medicine (95.6%), as well as the existence of this type of service in the national territory (96.4%). The great interest shown by 96.7% of the respondents in learning more about the subject highlights the importance of raising awareness, conducting further studies and promoting knowledge sharing in their daily clinical practice and research. The relationship between the national status of stem-cell therapy and veterinarians’ knowledge is influenced by limited regulation but growing professional interest and driven by private companies and professional organisations. This study contributes to a better understanding of the trends related to this topic in the context of veterinary practice in Portugal.

**Supplementary Information:**

The online version contains supplementary material available at 10.1186/s12917-025-04872-z.

## Background

In recent decades, there has been a notable advancement in the field of therapeutic strategies for complementary and regenerative veterinary medicine [1, 2]. The shift towards a preventive, early-detection, minimally invasive diagnostic and treatment paradigm has resulted in the development of personalised and targeted therapeutic strategies in both human and companion animal medicine [3, 4].

MSCs represent one of the most promising areas of therapeutic research. MSCs have “long been regarded as undifferentiated cells with the capacity for proliferation, self-renewal, the production of a large number of differentiated progeny, and regeneration of tissues” [5]. They are small cells that lack the phenotypic characteristics of adult tissues, but are capable of regenerating differentiated cells in their surrounding environment [6]. Their natural multipotency ability contributes to the regenerative capacity of MSCs, which is attracting worldwide attention for various applications [7, 8].

Cell-based therapies represent a promising avenue in the field of regenerative medicine, which aims to restore the normal form and function of the organism by harnessing its intrinsic biological machinery/capabilities. This option offers new hope for some refractory and/or chronic diseases with poor response to conventional therapies [9, 10]. In veterinary medicine, particular emphasis has been placed on MSCs, which are among the most widely recognised cell types [11]. A variety of different sources of SCs (Stem cells) have been identified including (i) embryonic tissue, which gives rise to embryonic SCs, (ii) fetal sources, such as fetus, placenta, umbilical cord and amniotic fluid, (iii) adult tissues, such as adipose tissue, bone marrow, muscle, skin and blood, and (iv) induced somatic differentiated cells which are generated in vitro by reprogramming somatic differentiated cells to a pluripotent state [4, 12].

The differentiation capacity of MSCs was initially thought to be the primary basis for their therapeutic effects in repairing and regenerating damaged tissues. However, contrary to this initial assumption, MSCs do not need to integrate or differentiate into specific tissue cells to exert their therapeutic effects, although the mechanisms of integration and differentiation are still thought to play a role in the therapeutic effects of MSCs [13]. As demonstrated in previous studies, MSCs have immunomodulatory and immunogenic therapeutic capabilities to regulate or alter the immune responses and positively influence immune function without rejection. They can be applied to a wide range of immune-mediated conditions through their capacity to modulate the inflammatory response. The mechanism in question exerts its function through the suppression of the immune system cells such as macrophages, dendritic natural killer cells and lymphocytes, thereby inducing anti-inflammatory subsets within both the innate and adaptive immune responses [14]. The immunogenic properties of SCs are pivotal in determining their potential for rejection following transplantation. This can be regarded as a concern and a limitation to their use, due to the potential for rejection, particularly in allogeneic (donor-derived) contexts. In vitro studies indicates that adipocytes, osteoblasts and chondrocytes, like undifferentiated MSCs, did not provoke a marked immune reactivity response, suggesting that allogeneic MSCs can be used for transplantation without a significant risk of rejection in therapeutic applications, due their immunomodulatory mechanism [15–17]. Despite the potential of these therapies, they are still not widely used in Portugal, which leads us to explore the reasons why. It is the authors’ hypothesis that the main reason for the poor use is a lack of knowledge or confidence of clinicians in recommending them.

The 2018’s survey conducted by the Federation of Veterinarians of Europe (FVE), reported that 87% of the Portuguese veterinarians income derived from the treatment of companion animals, despite the country having one of the lowest total incomes per veterinarian per year in Europe, with an average of less than <€20,000. This places Portugal in the second position, with a ranking of 87%, behind Russia, which has 89% in 2018 FVE survey (no data from 2023) [9, 18].

The majority of participating veterinarians in our survey, expressed interest and willingness to integrate SCs therapies into their practices, recognizing the potential benefits for their patients’ health and recovery. However, some responders highlighted a lack of access to specialised training and necessary resources for the effective implementation of these therapies [9].

In fact, the 2023 FVE survey revealed that the average European veterinarian dedicates 45 h per year to Continuing Professional Development (CPD) activities, marking an increase from the 40 h reported in the 2018 survey. In 2018, Portugal was among the ten countries with the lowest investment in continuous training, with an average of only 27 h spent on CPD per year [9, 18].

The objective of this study is to evaluate the knowledge of Portuguese veterinarians working in the companion animal sector and their interest/willingness to use/apply cellular therapies. The secondary objective is to determine the level of interest in facilitating access to MSCs and to develop a hypothetical model for implementing a national distribution network for this biological material.

## Methods

### Questionnaire

The target public of the questionnaire were the veterinarians working in companion animal clinic in Portugal.

The questionnaire was prepared in Portuguese language and consisted in 25 questions. The 25 questions were closed-ended or checkboxes, but in three of them, by filling in the option “Others” or “Yes” responders could give an answer that was not present among the listed options.

The survey covered the following three main topics: (i) veterinarians’ profile (five questions) aiming to know the veterinarian profile like time of practice work and main intervention area; (ii) Knowledge about cell therapies (10 questions) to discern the technical knowledge about cell therapies origins, therapeutic actions, advantages and disadvantages, and (iii) interest in future use and application (10 questions) in order to assess the perception of clinical practitioners and their future interest in application of these treatments as well as the importance of access to technical information about this cellular therapy (Supplementary file 1).

In the initial phase of internal validation, the questionnaire was evaluated by the quality department and statistical technicians for analysis. This pilot questionnaire was primarily tested and distributed exclusively through the authors and some veterinary practitioners (*n* = 10). Following this, the questionnaire was adapted in accordance with the comments and opinions obtained from these professionals. The pilot test did not contribute to the data collection for this study. The only useful outcome of this preliminary phase was the confirmation that the questionnaire was user-friendly.

Considering a population size of active members in the Portuguese Order of Veterinarians which is 7,114 active members, it can be inferred that 3800 practitioners work in companion animals’ area. Based on the population of active practicing members, the sample size represents 7.2% of the total number of active members (www.omv.pt/omv). The questionnaire was developed using an online platform (Google forms^®^).

In the second stage of the study, the validated questionnaire was disseminated among Portuguese veterinary groups on social networks and other online platforms for a period of 12 weeks, between November 2021 and January 2022. The questionnaire was anonymous, and the respondents were informed of the intention to use the data for research purposes. Additionally, the respondent was given the option to leave non-anonymous contact details for the purpose of receiving future information and updates regarding the work (*n* = 14). Furthermore, the questionnaire was approved by the ethics committee of the Vasco da Gama University School.

### Data processing and statistical analysis

All data were collected from the platform Google Forms^®^ and downloaded into a database (Microsoft^®^ Excel 2019, 16.78 version; Microsoft Corp., USA). To operationalize descriptive and inferential statistical analysis all the data was converted to R^®^ software (R^®^ 3.3.0 version, R Core Team, 2019).

A descriptive statistical analysis was employed to analyse the categorical data. A Chi-square test was applied to study the relationship between two variables in a contingency table, with a significance level of 5% (p-value < 0.05). The test is highly recommended to understand a relationship between categorical variables [19].

## Results

The data analysis included all 275 veterinary practitioners who responded to the survey and worked in Portugal. As no significant issues were identified during the internal validation process, the questionnaire remained consistent throughout the 12-week survey period.

The results are based on objective data and do not reflect any subjective evaluations.

### Veterinarians’ profile

Regarding the academic degree of the respondents, a similar frequency was found between Integrated Master in Veterinary Medicine (post-Bologna) and pre-Bologna Licentiate 46.5% (128/275) and 45.5% (125/275) respectively, 4.4% (12/275) with pre-Bologna Master and 3.6% (10/275) with PhD degree.

The experience of the participants, based on years of clinical practice, was distributed relatively evenly. The highest value of 21.5% (59/275), was observed among those with 11 to 15 years of clinical practice, while the lowest value, 8.7% (24/275) was observed among those with less than two years of practice. The remaining categories (2 to 5; 6 to 10; 16 to 20 and above 20) exhibit a similar distribution, with percentages ranging between 15% and 19%.

94.5% (260/275) of the respondents indicated that they are active in the practice of veterinary medicine with both canine and feline species. 13 respondents (4.7%) indicated that they were not currently engaged in clinical practice but had previously done so. Additionally, two respondents (0.8%) indicated that they had experience working with other species. The majority of veterinarians were based in urban areas, representing 59.6% (164/175) of the total sample. 76 respondents (27.6%) indicated that they had developed their practice in both rural and urban areas, while 35 respondents (12.7%) indicated that they had developed their practice in rural areas only. A total of 232 veterinarians reported their main activity as general clinical specialty area, representing 84.4%. The remainder reported their main activity in soft tissue surgery 10.2% (28/275), physical and rehabilitative medicine 1.4% (4/275), complementary and integrative medicine and emergency medicine 0.36% (1/275). Of the nine respondents (3.3%) who selected the “other” option, two indicated that their practice included dermatology, pathology and medical imaging, while one reported a focus on the pharmaceutical industry, reproductive medicine, and anaesthesia.

The statistical results obtained from the Chi-squared test demonstrate a significant association between the participants’ academic degree and their knowledge about the definition of SCs, their ability to differentiate into other cell types and their therapeutic applications in companion animals. The analysis of these parameters indicates that the observed differences between the groups are statistically significant, based on the defined significance level (p-value < 0.05) (Supplementary file 2).

The dependency between the respondent’s academic degree and what SCs are (considering their differentiation), the risk of the possibility of an immunogenic reaction using SCs, the knowledge of the immunomodulatory capacity of SCs and the possibility of preserving SCs demonstrated association with a p-value < 0.01 (Supplementary file 2).

### Knowledge about cell therapies

The majority of survey participants (90.9%, 250/275) reported familiarity with SCs and their therapeutic potential (94.5%, 260/275).

The knowledge about SCs was acquired through a variety of resources, predominantly in technical-scientific events 31.5% (82/260). In addition, a smaller percentage of respondents indicated that they had become aware of the subject matter through the scientific literature (28.8%, 75/260), from veterinary professionals or other sources (26.9%, 70/260), or via social media networks (6.2%, 16/260).

Seventeen individuals who participated in the survey, reported having contact through alternative information channels, including teaching contexts at the university level, research activities related to this topic, and social media platforms designed for human use and pregnancy.

**Fig. 1:**
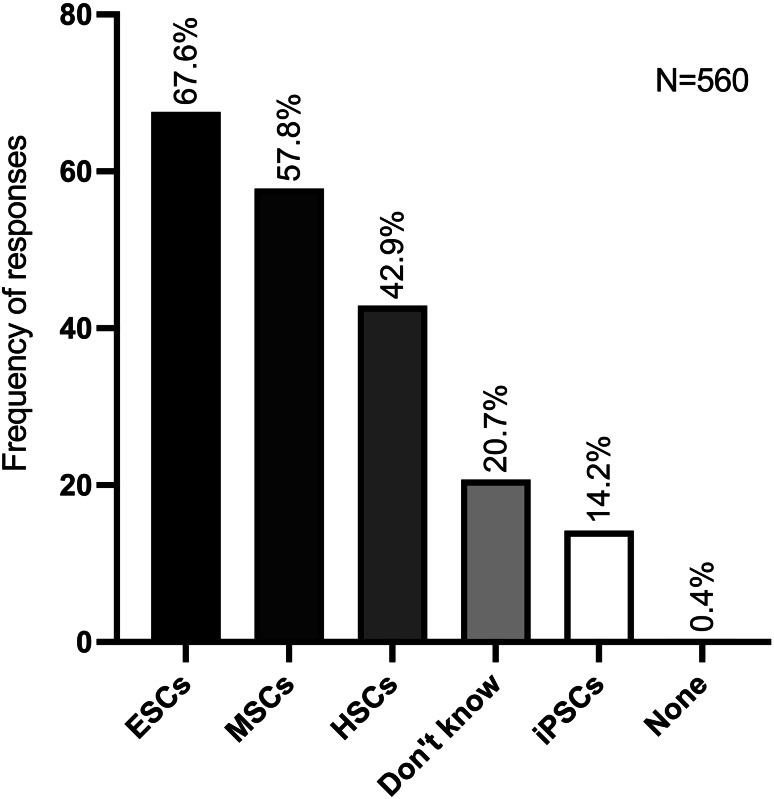
Frequency of responses about the different types of stem cells according to their origin in a total of 275 respondents. ESCs, Embryonic Stem Cells; MSCs, Mesenchymal Stem Cells; HSCs, Hematopoietic Stem Cells; iPSCs, Induced Pluripotent Stem Cells. (more than one option could be chosen)

In response to the question regarding the diverse origins of SCs, the majority of respondents (67.6%) selected embryonic, with 186 responses. This was followed by mesenchymal (57.8%), with 159 selections, haematopoietic (42.9%), with 118 choices, and induced pluripotency (14.2%), with 39 preferences. The survey results indicate that 57 participants (20.7%) were uncertain or lacked knowledge in this regard, while one respondent (0.4%) selected none of the available options (Fig. 1). It is important to note that, in this section, participants were allowed to select more than one option in questions 10 and 14 (Supplementary file 1).

A total of 253 respondents (92% of the total number surveyed) indicated that SCs can be preserved. Conversely, 17 respondents (6% of the total number surveyed) indicated that they were unsure if SCs could be preserved. Five respondents (2% of the total number surveyed) stated that preservation of SCs was not possible.

The data collected indicated that 22.5% of respondents believed that SCs use does not result in immunogenic reactions (62/275). In contrast, 5.1% (14/275) of respondents indicated that immunogenic reactions are possible, while 72.4% (205/275) were unsure or unaware of this possibility.

Regarding the immunomodulatory ability 53.5% (147/275) claim to recognize this effect while 45.1% (124/275) are not aware and 1.4% (4/275) won’t believe in their immunomodulatory potential. When inquired about the tissues from which MSCs can be sourced, 38.5% (106/275) responded with a lack of knowledge.

**Fig. 2:**
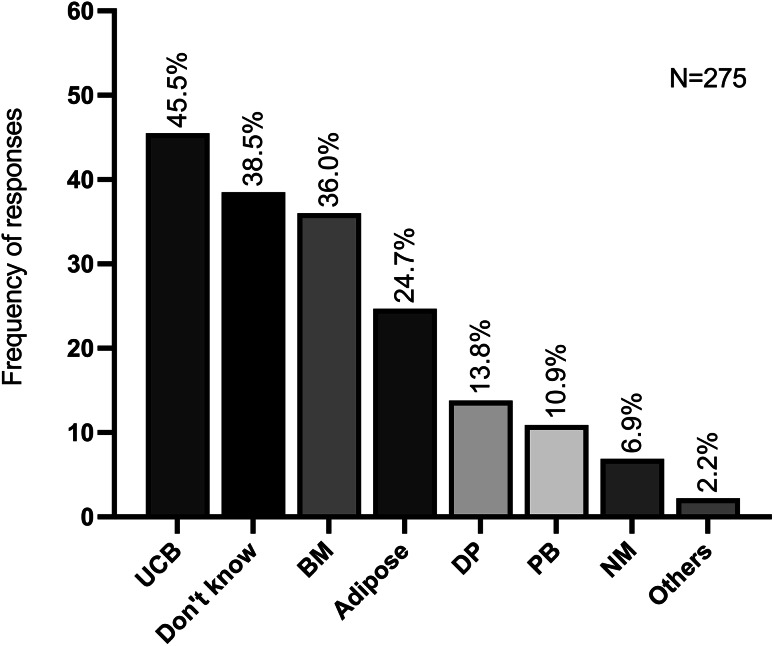
Frequency of responses regarding different tissue sources for obtaining MSCs. UCB, Umbilical Cord Blood; BM, Bone Marrow; DP, Dental Pulp; PB, Peripheral Blood; NM, Nasal Mucosa. (more than one option could be chosen)

Of the remaining 150 responses the most frequently selected source was umbilical cord blood with 45.5% (125/275) of respondents indicating this as a potential source. This was followed by bone marrow (36.0%; 99/275) and adipose tissue (24.7%; 68/275). Furthermore, a smaller number of respondents selected for dental pulp (13.8%; 38/275), peripheral blood (10.9%; 30/275), nasal mucosa (6.9%; 19/275) and “other” (2.2%; 6/275) tissues as potential sources for obtaining MSCs (Fig. 2).

A total of 56.7% (156/275) of the participants indicated that they were unaware of the use of cell-based therapies utilising SCs in the treatment of veterinary companion animals. Of the 119 positive responses (43.3%) regarding the utilisation of SCs therapies in veterinary medicine, 93 respondents specified the pathologies for which they were aware of the application. The collected data demonstrated that the clinical areas most frequently referenced by the participants were related to orthopaedic conditions (*n* = 71) and dental or oral diseases (*n* = 21). Among the initial categories, the most frequently mentioned affections were articular (51 times), such as inflammatory and degenerative joint diseases including osteoarthritis (20 times). Regarding oral diseases, feline chronic gingivostomatitis (FCGS) was identified on 17 occasions and periodontal diseases on four occasions.

The next clinical situation cited 17 times concerns to neurologic conditions mostly in spinal cord lesions (6/17) followed by regenerative medicine named 16 times in particular tissue healing (12/16) and peripheral nerve regeneration (2/16). Nephrology was mentioned 10 times, nine of those in chronic kidney disease (CKD). The dermatological pathology most discussed was atopic dermatitis (6/7).

**Fig. 3:**
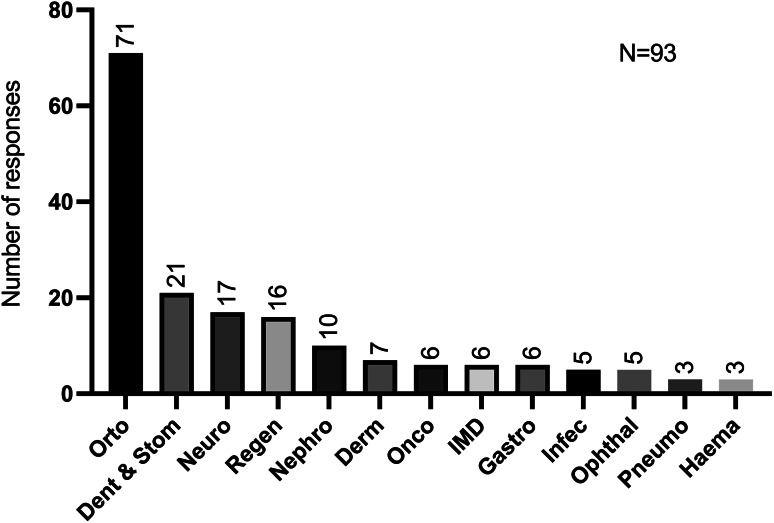
Number of responses about participants’ knowledge of SCs application by clinical area. Orto, Orthopaedic; Dent & Stom, Dentistry and Stomatology; Neuro, Neurology; Regen, Regenerative; Nephro, Nephrology; Derm, Dermatology; Onco, Oncology; IMD, immune-mediated diseases; Gastro, Gastroenterology; Infec, Infectious diseases; Ophthal, Ophthalmology; Pneumo, Pneumology and Haema, Haematology. (more than one answer was possible)

It is worth noting that six respondents did not provide detailed clinical information regarding immune-mediated diseases, only specifying the main area. The most frequently referred immune-mediated diseases potentially treated with SCs were FCGS and inflammatory bowel disease, with 17 and 6 mentions, respectively. Oncological and gastrointestinal diseases were mentioned six times each of them, while infectious and ophthalmologic diseases were referred five times, both. The domain of pneumology and haematological disorders was mentioned only three times, indicating a relatively limited scope of attention. (Fig. 3).

Table with detailed citations is available as supplementary material (Supplementary file 3).

In this section, the statistical results obtained from the Chi-squared test did not show statistical significance, with the p-value exceeding the established significance level (p-value < 0.05).

### Interested in future use and application of stem cells

With regard to future interest, 46.2% (127/275) of respondents expressed positive attitudes towards the proposed therapeutic option, while 8.4% (23/275) indicated reservations. As anticipated, 45.5% (125/275) of veterinarians indicated a lack of knowledge regarding the acceptance of SCs.

The majority of veterinarians who participated in this survey expressed reservations about the opinion and receptiveness of the tutor. Specifically, 51.6% (142/275) of the respondents indicated doubt, while 39.6% (109/275) expressed belief in positive acceptance by tutors. The remaining 8.7% of respondents indicated that cell-based therapies are not well accepted. Nevertheless, practitioners demonstrate a considerable deficit in knowledge regarding this biological product. Specifically, 57.5% (158/275) indicate that their understanding of SCs is merely superficial, 92.7% (255/275) report a lack of proficiency in its application, and 90.5% (249/275) have no prior experience with this therapeutic option. Among the 9.5% (26/275) respondents who reported previous experience with cell therapies, 13 were employed in clinical practice, 7 in soft tissue surgery, 4 in physical medicine and rehabilitation, and clinical pathology and reproductive medicine, with 1 response each. The investigation revealed no associations between the groups of respondents who had used cell therapies and those who had not, regarding the veterinarian profile and knowledge of this therapies type parameters.

**Fig. 4:**
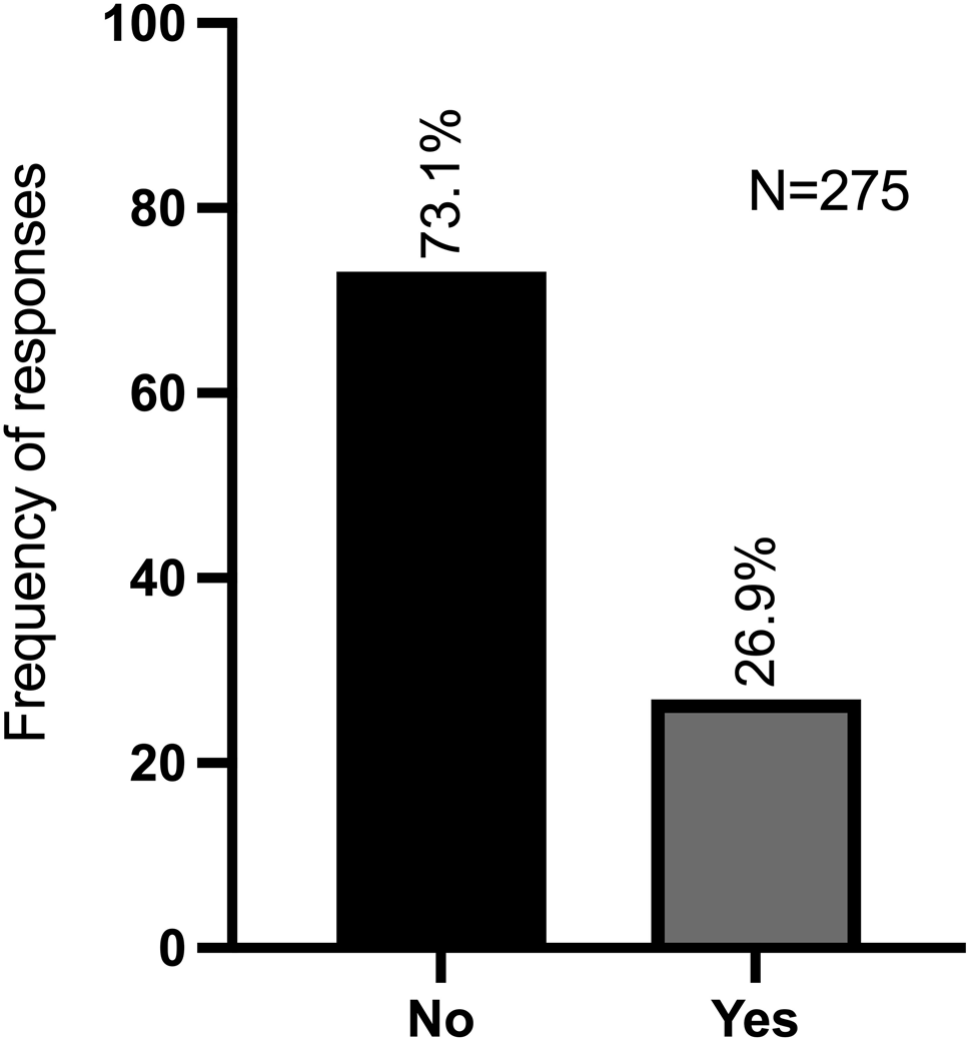
Frequency of participants who have already considered the use of cellular therapies

Of the 275 veterinarians surveyed, 201 (73.1%) have never considered the use of SCs, as illustrated in Fig. 4. Among the 74 (26.9%) respondents who have contemplated the utilisation of SCs as an alternative or complementary treatment option for their patients, 61 specified up to three clinical scenarios, in which they would have preferred to use them. A total of 138 clinical scenarios were collected.

**Fig. 5:**
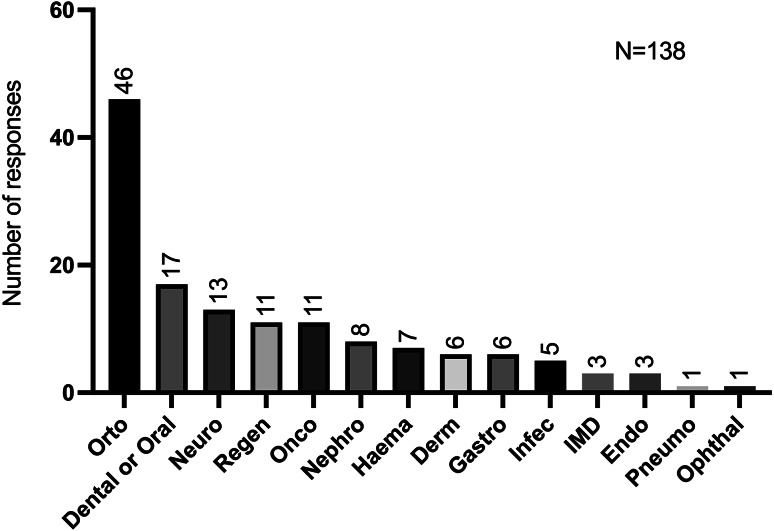
Clinical areas most mentioned by participants related to the desire to apply SCs. Orto, Orthopaedic; Neuro, Neurology; Regen, Regenerative; Onco, Oncology; Nephro, Nephrology; Haema, Haematology; Derm, Dermatology; Infec, Infectious diseases; IMD, immune-mediated diseases; Endo, Endocrinology; Pneumo, Pneumology and Ophthal, Ophthalmology (up to three possible answers per participant)

The responses obtained were classified in order to facilitate interpretation. The category with the highest number of references was orthopaedics, with 46 references, followed by dentistry & stomatology, and neurology with 17 and 13 occurrences respectively. Regenerative medicine and oncology were the fourth most frequently mentioned category, with 11 citations. These were particularly focused on tissue healing but also in oncological conditions such as leukaemia, squamous cell carcinoma (SCC) and canine lymphoma. The least frequently cited area was nephrology, with only 8 statements, specifically addressing CKD. 7 references were made to haematologic diseases, particularly bone marrow aplasia and anaemia, with the intention of using SCs. As illustrated in Fig. 5, dermatology and gastroenterology were reported with lesser frequency, with only six occurrences each. Infectious diseases were reported 5 times, while immune-mediated and endocrinology domains were reported 3 times. Pneumology and ophthalmology were each cited on a single occasion.

Table with detailed data and clinical situations is available as supplementary material (Supplementary file 4).

A statistically significant *p* < 0.05 association was identified between academic degree and the use of SCs and with their having already use SCs and the consideration of their use in chronic or refractory clinical conditions as a complement to conventional therapy. Dependency was observed between the academic degree and the perception of the surveyed individuals regarding the acceptance of the use of SCs by the veterinary medical community presenting a p-value < 0.01 (Supplementary file 2).

## Discussion

The present study employs data obtained from a questionnaire completed by 275 veterinary practitioners, to evaluate the general and practical knowledge of cell therapies held by veterinary practitioners in Portugal, as well as to ascertain their future perspectives on this issue.

The objective was to determine the extent to which professionals adhere to and repose trust in the utilisation of cell-based therapies.

In humans, as well as in veterinary medicine, chronic injuries and pathologies result in significant economic burden, with costs reaching thousands of euros or dollars per year.

Additionally, some injuries and diseases are also misdiagnosed, which contributes to an increase in clinical cases and a worsening of the condition. The need to improve the processes of recovery and re-establishment of functionality has resulted in a substantial expansion in the regenerative medicine domain using SCs [10].

The immunogenicity of SCs, and particularly that of pluripotent stem cells (PSCs), such as embryonic stem cells (ESCs) and induced pluripotent stem cells (iPSCs), is a critical consideration for their therapeutic application. While these cells demonstrate the capacity for differentiation into a range of cell types, their immunogenic properties have the potential to result in immune rejection following transplantation.

Regarding the immunogenicity and tumourigenicity, it has been demonstrated that certain iPSC-derived cells have the capacity to elicit immune responses, even in autologous transplantation contexts. This potential has been attributed to genetic or epigenetic alterations that may occur during the reprogramming process or the expression of novel antigens. It is important to note that both ESCs and iPSCs carry a risk of teratoma formation if undifferentiated cells are transplanted, raising significant safety concerns. Conversely, some ESC-derived tissues exhibit immune-privileged properties, reducing the risk of rejection, however, this depends on the differentiation state and tissue type derived from PSCs. In order to mitigate the occurrence of immunogenicity, current strategies include the implementation of genetic modifications to downregulate MHC expression, the utilisation of immunosuppressants, and the induction of immune tolerance through various protocols [20–22].

A critical distinction in stem-cell therapy is the use of autologous (self-derived) versus allogeneic (donor-derived) cells.

The autologous therapies have been shown to reduce the risks associated with immune rejection and typically do not require immunosuppression. These include treatments such as adipose-derived MSCs or autologous transplants for cancers like leukaemia. Nevertheless, despite their patient-specific nature, iPSCs may still pose safety risks due to the potential for immunogenicity or genomic instability.

Allogeneic therapies, on the other hand, offer an alternative source of cells and have been shown to be effective in cases where patient cells are deemed unsuitable. Nevertheless, these transplants carry a higher risk of immune rejection and frequently require immunosuppressive therapy [20–22]. A number of studies on animals, including cats, have demonstrated that allogeneic adipose SCs result in delayed immune responses when compared with autologous treatments. Despite some non-life-threatening secondary reactions as a consequence of the rate flow administration of the SCs treatments (e.g., limb oedema, increased respiratory rate, vomiting and diarrhea) that resolved spontaneously, the safety of both autologous and allogenic cell sources when administered systemically appears to be assured [23–28].

Regarding the optimal timing for treatment is contingent upon the specific stage of the injury (acute, sub-acute or chronic) and the intended therapeutic objective (control of inflammation, regeneration or remodelling). The nature of the tissue also determines the most appropriate administration route, with local strategies being favoured for specific tissues and systemic approaches being recommended for more generalised conditions [12, 29–32]. The contents of Supplementary file 5 provide a concise summary of the application time and delivery method of stem-cell therapies.

In order to define multipotent SCs, the International Society for Cellular Therapy has established three specific criteria for MSCs. Characterized with a fibroblast-like shape, the first criteria is for these cells to be plastic adherent cells that proliferate in vitro. The second criteria is that the cells must express specific surface markers and lack expression of others by flow cytometry. Thirdly, the cells must be differentiated to osteoblasts, chondrocytes and adipocytes within an in vitro environment [33, 34].

In 2008 the Food and Drug Administration (FDA) conclude that “*products derived from stem cells*, *whether embryonic or adult in origin*, *are simply a subclass of the broader category of cellular therapies and can be addressed similarly from the standpoint of regulatory authorities and guidance currently in place in the USA*”. Products derived from SCs (from all sources) are consider somatic cellular therapies regulated by the Public Health Act [35].

During December 2024, FDA has approved 43 human cellular and gene therapy products from the Office of Tissues and Advanced Therapies listed on the FDA website [36].

Nevertheless, the market for cell therapies for veterinary use is more restricted and still in a state of development. The majority of companies operating within the field of veterinary SCs research are primarily oriented towards the provision of cryopreservation and personalised application services, as opposed to the large-scale marketing of ready-to-use products.

The regulatory framework pertaining to cell therapies within Europe is overseen by the European Medicines Agency (EMA), through the Committee for Advanced Therapies (CAT). The CAT’s primary responsibility is to ensure the quality, safety and efficacy of products prior to their introduction into the market. Also, the regulation status of cell therapies for veterinary use varies between European countries. In many cases, therapies are classified as experimental or customisable treatments, requiring only specific authorisation from national veterinary authorities and a guarantee of compliance with good manufacturing practice standards. Despite the presence of numerous companies specialising in this field, there is currently no complete list of companies that has been recognised by the EMA [37].

In Portugal, the use of SCs in veterinary medicine is also emerging but is still limited in terms of exclusively commercialising companies. Pioneering companies in this field include *Vetherapy* and *StemVet Portugal*, which offer cryopreservation services and the application of cell therapies.

Also in the year 2020, international regulatory considerations for the development of stem cell-based veterinary medicinal products are published. These considerations include the establishment of safety assessment guidelines, which facilitate the development and availability of stem cell-based products. This contributes to an improvement in the treatment and quality of life of companion animals suffering from chronic or incurable diseases [38].

In human medicine, it was concluded that European regulations on the therapeutic use of stem cells are clear, particularly concerning the development of Cell Therapy Medicinal Products, and developers are advised to consistently consider essential regulatory criteria throughout the process, such as (1) “SCs are most often considered as Advanced Therapy Medicinal Products in EU and are classified by the Committee for Advanced Therapies (CAT)”; (2) “Stem cells are one of the most complex medicinal products”; (3) “Starting materials have a major impact on quality”; and (4) “The clinical development should comply with updated EU regulation” [39].

The paradigm shift has served to reinforce the promising capacity of these therapies in both human and animals subjects.

Many human diseases including cancer, neurodegenerative disorders and dermatological conditions such as oral lichen planus have counterparts in veterinary medicine, highlighting the need for further research into cell-based therapies and the validation of animal disease models. Companion animals, such as dogs and cats, are particularly valuable in this context, as they naturally develop diseases that closely resemble human conditions in terms of pathophysiology and aetiology, including tumours, neurological, immunological and cardiovascular disorders. Their shared living environment with humans and exposure to similar environmental factors enhances their relevance as translational models, while their genetic diversity provides a more accurate representation of human population variability compared to traditional laboratory animals [40–42].

This aspect facilitates a more precise evaluation of the individual response to diseases and treatments [43, 44].

A number of animal studies have demonstrated the efficacy of cell-based therapies, particularly MSCs, in treating a variety of conditions, including degenerative joint diseases such as osteoarthritis, immune-mediated diseases such as FCGS and inflammatory bowel disease (IBD), periodontal disease, arthritis, encephalomyelitis, various dermatological conditions such as wounds, atopic dermatitis and erythematous lupus, and neurodegenerative diseases. Furthermore, these therapies have demonstrated efficacy in managing musculoskeletal and systemic conditions, including diabetes and kidney failure, underscoring their potential as a valuable strategy for promoting recovery and disease control in affected animals [3, 4, 8, 45].

The objective of this survey was to ascertain whether veterinarians are aware of the potential benefits and applications of cell-based therapies using SCs. Predictably, the majority of respondents did not consider the potential use of SCs in the treatment of a diversity of diseases and/or clinical situations, as can be seen in Fig. 4.

The statistically significant associations revealed by the chi-square test suggest that higher academic qualifications correlate with more informed and favourable perceptions of stem cells, indicating that formal education plays a gatekeeping role in access to cutting-edge biomedical concepts. A critical analysis of this finding points to a deeper, systemic issue: the disparity in knowledge and attitudes towards SCs technologies is not merely a matter of individual interest or exposure but is structurally rooted in educational pathways.

The observed association between academic degree and knowledge of SC differentiation and therapeutic use suggests that awareness and understanding of cutting-edge biomedical innovations is still largely confined to higher education environments.

It is imperative to recognise that this underscores a knowledge stratification issue within the veterinary domain. Those lacking advanced degrees may encounter challenges in accessing or comprehending transformative technologies, such as stem cell therapies. This situation gives rise to significant questions regarding knowledge dissemination and accessibility.

The finding suggests that without broader educational efforts, advancements may fail to reach veterinary clinical practice. It emphasises the necessity for ongoing education and professional training to facilitate the dissemination of these innovations beyond the academic and specialist fields.

The highly significant associations (p-value < 0.01) found between academic qualifications and respondents’ understanding of advanced SC-related concepts, including the risk of immunogenic reactions, the immunomodulatory properties of SCs, and the feasibility of preserving SCs for future use, suggests that a deeper academic foundation promotes a more nuanced understanding of the complexities and technical aspects of SCs science.

The correlation between academic qualification and increased understanding of advanced concepts such as immunogenic risk, immunomodulation and cryopreservation suggests that current educational structures may inadvertently restrict access to critical, practice-relevant knowledge.

The insight is that beyond scientific breakthroughs, the translation into everyday veterinary practice will depend on breaking down educational gaps by embedding advanced content into broader curricula and creating targeted, accessible learning pathways for all levels of veterinary professionals.

The participants demonstrated a comprehensive understanding of the origin and potential of SCs. However, their comprehension of cell-based therapies exhibited considerable variability and heterogeneity.

The validity of this assertion is substantiated by the finding that among the 119 veterinarians who claimed competence in the domain of stem cell utilisation in veterinary medicine, a comparatively smaller proportion of 25 had actually applied stem cells as an alternative or complementary therapy in their clinical practice.

Findings indicates that veterinarians are open to the use of stem cells as a complementary or alternative therapy, with the majority of interest focused on orthopaedic conditions, particularly osteoarthritis, joint pathologies, and musculoskeletal disorders. This suggests that veterinarians are more likely to consider stem cell therapies when there is clear evidence or familiarity with their effectiveness in specific conditions.

A critical evaluation indicates that while SC therapies are being investigated in a variety of domains, distinct differences emerge with regard to clinical readiness and practical application. The most promising areas for immediate use appear to be orthopaedics, dental/oral conditions, and regenerative medicine, whereas further research, validation, and standardisation may be required for neurology and oncology to overcome existing clinical challenges. This observation underscores the necessity for targeted research and education in these specialised domains to facilitate the safe and effective integration of stem cell therapies in veterinary practice.

The results also demonstrate an increasing interest among various specialties and conditions such as chronic kidney disease (CKD), atopic dermatitis, and IBD exemplifying the versatility for both acute and chronic conditions. However, the less frequent mention of specialties such as nephrology and ophthalmology raise the question of whether SC therapies are under-utilised or there is insufficient research and evidence in these areas. The results, summarised in Supplementary file 4, suggest a growing openness among veterinarians to use, although the adoption of this approach may vary according to factors such as clinical experience and the availability of research and evidence. The findings should be interpreted as exploratory, with a careful approach due to the relatively small sample size of the PhD group (n = 10) compared to the non-PhD group (n = 265).

The wide range of conditions cited highlights the versatility of SCs and their ability to treat both acute and chronic conditions. The results indicate a growing awareness and interest among veterinarians in integrating SCs therapies into their practices. However, they also highlight the need for further clinical trials, standardisation of protocols and regulatory frameworks to ensure safety and efficacy. The variation in interest across specialties may reflect differences in disease prevalence, existing therapeutic gaps, or the current level of research and evidence supporting SCs applications. This discrepancy can be attributed to several factors, including limited access to specialised education, scientific resources, and clinical protocols which influence the willingness and ability to integrate innovative therapeutic modalities into practice, essential for the advancement of veterinary medicine. Among the respondents, 147 demonstrated knowledge of SCs immunomodulatory functions, and 22 have already applied them in clinical practice, particularly for immune-mediated conditions such as FCGS and IBD, which appear to be common in veterinary caseloads and represent promising targets for these emerging therapies.

## Conclusion

The findings from this study demonstrate an increasing interest among Portuguese veterinarians in the application of SCs therapies across various domains of veterinary medicine, particularly in orthopaedics, neurology, dentistry, and regenerative medicine. While there is a general acknowledgement amongst professionals of the therapeutic potential of stem cells, particularly in the context of chronic and refractory conditions, a discernible gap persists between interest and implementation. A statistically significant correlation between academic qualifications and the utilisation of SCs suggests that higher educational attainment influences both awareness and practical application of these innovative therapies. This underscores the pivotal function of advanced training in equipping healthcare practitioners with the requisite knowledge and confidence to integrate SCs into standard clinical practice.

The survey results indicate that veterinarians with higher academic qualifications are more likely to perceive SCs therapies as a viable and accepted practice in veterinary medicine. This perception is likely influenced by their greater exposure to scientific advancements, literature, and academic discourse. This underscores the critical need to ensure that all veterinarians are adequately informed about SCs and their diverse therapeutic potential, particularly their regenerative and immunomodulatory properties. While awareness of SC applications is growing, the results also highlight a significant demand for more comprehensive educational and informational resources to support their responsible and effective use. It is noteworthy that individuals with the confidence to advise on SC-based therapies tend to have between 16 and 20 years of clinical experience, primarily acquired in urban settings where access to continuing education and advanced treatments may be more readily available.

The FVE 2023 survey shows that 55% of the veterinarians expect the sector to grow and 25% expect the number of veterinarians to increase, highlighting the need for continued investment in technical training and knowledge expansion to effectively implement innovative therapies alongside conventional treatments.

However, the current FVE-CPD satisfaction rating of 6.4 out of 10 (with 0 being completely dissatisfied and 10 completely satisfied) reflects a demand for improved access to technical training and evidence-based clinical protocols. This highlights the urgent need for coordinated action by educational institutions, veterinary associations, and regulators to foster a supportive and ethically sound environment for SCs research and application. Future efforts should thus concentrate on the implementation of structured continuing education programs, clearer regulatory and ethics guidelines and the development of outreach initiatives aimed at demystifying SCs for both professionals and pet owners. By taking such actions, the veterinary community can elevate animal welfare and responsibly navigate the evolving landscape of regenerative medicine. The findings of this study provide a robust foundation for future initiatives aimed at strengthening the understanding and application, improving patient outcomes and advancing of SCs in the veterinary field.

## Electronic supplementary material

Below is the link to the electronic supplementary material.

Supplementary Material 1

## Data Availability

Data is provided within the manuscript or supplementary information files.

## Abbreviations

SCs: Stem cells
MSCs: Mesenchymal stromal cells
FCGS: Feline Chronic Gingivostomatitis
CKD: Chronic Kidney Disease
SCC: Squamous Cell Carcinoma
FVE: Federation of Veterinarians of Europe
PSCs: Pluripotent Stem Cells
ESCs: Embryonic Stem Cells
iPSCs: Induced Pluripotent Stem Cells
FDA: Food and Drug Administration
EMA: European Medicines Agency
CAT: Committee for Advanced Therapies
IBD: Inflammatory Bowel Disease
CPD: Continuing Professional Development
