# Supplementary material for: Knowledge of companion animals’ practitioners on stem-cell based therapies in a clinical context: a questionnaire-based survey in Portugal
Source: BMC Vet Res. 2025 Jul 24;21:487. doi: 10.1186/s12917-025-04872-z (PMC12288361; doi:10.1186/s12917-025-04872-z)
Supplement: Supplementary file 1 — Supplementary Material 1 [file 12917_2025_4872_MOESM1_ESM.zip › 12917_2025_4872_MOESM1_ESM/12917_2025_4872_MOESM1_ESM.pdf]

Knowledge about cellular therapies of the medical-veterinary class in Portugal. -- This questionnaire is addressed to veterinary professionals who have already performed and/or perform their activity in companion animal sector. –

This work directed to veterinary professionals aims to evaluate the knowledge about cellular therapies and their clinical application.

This survey is part of a Phd project of the University of Trás-os-Montes e Alto Douro (UTAD) in collaboration with the University School Vasco da Gama (EUVG).

It consists of three parts: "Profile of the Veterinarian", "Knowledge about cell therapies" and "Interest in future use".

The questionnaire consists of 25 questions and takes about 5 minutes to be answered.

All answers given are anonymous and will be treated with secrecy, we asked to respond honestly in order to obtain valid and representative conclusions.

If you have any questions about the questionnaire do not hesitate to contact via email - Rafael.lopes@euvg.pt.

PART I - PROFILE OF VETERINARIAN DOCTOR

1. What is your academic degree in Veterinary Medicine? Select the highest.

- Phd (Veterinary sciences or other)
- Integrated Master in Veterinary Medicine (post-Bologna)
- Master's Degree in Veterinary Medicine (pre-Bologna)
- Degree in Veterinary Medicine (pre-Bologna)

2. How many years of clinical practice as Veterinarian Doctor you have?

- less than 2
- 2 to 5
- 6 to 10
- 11 to 15
- 16 to 20
- More than 20

3. Do you practice in companion animal's clinic specifically in dog and/or cat species?

- Yes, in both of
- Yes, only in dog
- Yes, only in cat

- Not currently, but I have
- No, I practice in another species(s)

4. In what type of geographical area did you exercised/exercise your clinical practice?

- Rural
- Urban
- Both

5. What is your main area of intervention?

- Clinical
- Surgery (general/soft tissue/orthopedic/neurological)
- Emergencies
- Physical Medicine and Rehabilitation
- Complementary and integrative medicine
- Others

5.1. If other has answered, please indicate:

PART II - KNOWLEDGE OF CELLULAR THERAPIES

6. Do you have any knowledge of what stem cells are?

- Yes
- No

7. Have you heard/know the therapeutic potential of stem cells?

- Yes
- No

7.1 If so, please indicate where you have obtained information about the subject

- scientific literature
- Technical-scientific events (Congress/Webinar/Course)
- Social media
- Through other professionals (veterinary or other)
- Others

7.2 If you have answered others, indicate which/which one/s

8. Based on your knowledge stem cells are:

- Differentiated cells

- Undifferentiated cells
- Don't know

9. Stem cells have the ability to differentiate into other cell types?

- Yes
- No
- Don’t know

10. There are different types of stem cells according to their origin. Enter the ones you know

- Mesenchymal cells
- Embryonic cells
- Induced pluripotent cells
- Hematopoietic cells
- None of the above cells
- Don’t know

11. The use of mesenchymal stem cells is risky due to the possibility of immunogenic reaction

- Yes
- No
- Don’t know

12. One of the characteristics of stem cells is their immunomodulatory capacity

- Yes
- No
- Don’t know

13. Based on your knowledge, is it possible to conserve stem cells?

- Yes
- No
- Don’t know

14. As regards the origin of mesenchymal stem cells, which tissues can they be obtained from?

- Peripheral blood
- Umbilical cord blood
- Bone marrow
- Adipose tissue
- dental pulp
- Nasal mucosa
- Others
- Don’t know

15. Did you know about the use of stem cells in the treatment of diseases in veterinary medicine?

- Yes
- No

15.1. If yes, please indicate which disease/s:

PART III - INTEREST IN FUTURE USE

16. Do you think that the use of stem cells is well accepted by the veterinary medical community?

- Yes
- No
- Don't know

17. Do you think that the use of stem cells is well accepted by tutors?

- Yes
- No
- Don't know

18. What is your perception about stem cells?

- They are the future of cell therapy
- I don't believe in this therapeutic option
- I know little about the subject
- It's just a business strategy

19. Do you feel have sufficient knowledge to advise and apply this therapy in clinical practice?

- Yes
- No

20. Have you ever used stem cells as a treatment option?

- Yes
- No

21. Have you considered the use of cell therapies as an alternative/complement to conventional therapies in the treatment of refractory diseases to conventional treatments and/or chronic diseases?

- Yes
- No

22. Name up to 3 clinical situations where you have used/desired to use stem cells as a therapeutic option.

23. After this questionnaire, are you curious to know more about the subject?

- Yes
- No

24. Do you consider this subject to be relevant in the veterinary medicine area?

- Yes
- No

25. Do you think the existence of this type of service in Portugal is important?

- Yes
- No

Thank you very much for the time you have taken to respond to this questionnaire. If you want to, please leave your comments and/or suggestions. If you desired to be contacted in the scope of this work, enter your email (not anonymous) or contact us through the email Rafael.lopes@euvg.pt
