# Supplementary material for: Knowledge of companion animals’ practitioners on stem-cell based therapies in a clinical context: a questionnaire-based survey in Portugal
Source: BMC Vet Res. 2025 Jul 24;21:487. doi: 10.1186/s12917-025-04872-z (PMC12288361; doi:10.1186/s12917-025-04872-z)
Supplement: Supplementary file 1 — Supplementary Material 1 [file 12917_2025_4872_MOESM1_ESM.zip › 12917_2025_4872_MOESM1_ESM/12917_2025_4872_MOESM3_ESM.docx]

**Supplementary File 5**

Summary of timing of application and delivery method of stem cell-based therapies.

| **Medical** | **Timming** | **Therapeutic goal** | **Delivery method** |
| --- | --- | --- | --- |
| **Cardiovascular Injuries** (e.g., myocardial infarction, MI) | Acute (0–48h) | Reduce inflammation, promote angiogenesis | Systemic or local (e.g., intramyocardial) |
|  | Sub-acute | Enhance Vascular regeneration | Systemic or intramyocardial injection |
|  | Post-MI (4–30 days) | Promote remodelling, angiogenesis, reduce fibrosis | Intracoronary, intramyocardial, or epicardial (e.g., cell sheet, spray) |
| **Neurological injuries** | Early | Limit inflammation and degeneration | Local, systemic or scaffold-assisted |
|  | Later | Promote axonal growth, neural regeneration | Local, systemic or scaffold-based/ |
| **Muscular injuries** | Acute (≤7 days) | Reduce inflammation and fibrosis | Local or systemic (e.g., intramuscular) |
|  | Sub-acute (2–4 weeks) | Stimulate regeneration and angiogenesis | Local or systemic |
| **Bone & craniofacial injuries** | Sub-acute to late | Matrix formation, vascularization, remodelling | Scaffold-based (e.g., hydrogels) |
| **Skin injuries** | Acute | Reduce inflammation, stimulate angiogenesis | Topical (gel/spray) or local injection |
|  | Sub-acute (1–2 weeks) | Promote epithelial repair | Topical or local |
| **Tendon & ligament injuries** | Sub-acute to late | Prevent fibrosis, support regeneration | Scaffold-based or direct local application |
