# Supplementary material for: Knowledge of companion animals’ practitioners on stem-cell based therapies in a clinical context: a questionnaire-based survey in Portugal
Source: BMC Vet Res. 2025 Jul 24;21:487. doi: 10.1186/s12917-025-04872-z (PMC12288361; doi:10.1186/s12917-025-04872-z)
Supplement: Supplementary file 1 — Supplementary Material 1 [file 12917_2025_4872_MOESM1_ESM.zip › 12917_2025_4872_MOESM1_ESM/12917_2025_4872_MOESM4_ESM.docx]

| **Clinical area** | **Total** | **Condition** |  |
| --- | --- | --- | --- |
| Orthopaedics | 71 | Articular diseases | 51 |
|  |  | Musculoskeletal disorders | 4 |
|  |  | Orthopaedic Surgery | 4 |
|  |  | Bone fractures | 2 |
|  |  | No response | 10 |
| Dentistry and Stomatology | 21 | Feline chronic gingivostomatitis | 17 |
|  |  | Periodontal disease | 4 |
| Neurology | 17 | Spinal cord injury | 6 |
|  |  | Neurodegenerative disorders | 3 |
|  |  | Intervertebral disc disease | 2 |
|  |  | Degenerative myelopathies | 1 |
|  |  | No response | 5 |
| Regenerative Medicine | 16 | Tissue healing | 12 |
|  |  | Peripheral nerve | 2 |
|  |  | Perianal fistula | 2 |
| Nephrology | 10 | Chronic kidney disease | 9 |
|  |  | Acute kidney disease | 1 |
| Dermatology | 7 | Atopic dermatitis | 6 |
|  |  | No response | 1 |
| Oncology | 6 | Leukaemia | 1 |
|  |  | No response | 5 |
| Immunology | 6 | Immune diseases | 6 |
| Gastroenterology | 6 | Inflammatory bowel disease | 6 |
| Infectious diseases | 5 | Canine distemper | 3 |
|  |  | Viral disease | 1 |
|  |  | No response | 1 |
| Ophthalmology | 5 | Keratoconjunctivitis sicca | 2 |
|  |  | Corneal ulcer | 1 |
|  |  | No response | 2 |
| Pneumology | 3 | Asthma | 1 |
|  |  | Pulmonary fibrosis | 1 |
|  |  | No response | 1 |
| Haematology | 3 | Bone marrow aplasia | 2 |
|  |  | Haematological disorders | 1 |

**Supplementary File 3**

Summary of responses provided by the 93 participants on the therapeutic applications of stem cells (question 15.1) categorized by clinical area and specific medical conditions.
