# Supplementary material for: Knowledge of companion animals’ practitioners on stem-cell based therapies in a clinical context: a questionnaire-based survey in Portugal
Source: BMC Vet Res. 2025 Jul 24;21:487. doi: 10.1186/s12917-025-04872-z (PMC12288361; doi:10.1186/s12917-025-04872-z)
Supplement: Supplementary file 1 — Supplementary Material 1 [file 12917_2025_4872_MOESM1_ESM.zip › 12917_2025_4872_MOESM1_ESM/12917_2025_4872_MOESM5_ESM.docx]

**Supplementary File 4**

| **Medical area** | **Total** | **Condition** |  |
| --- | --- | --- | --- |
| Orthopaedics | 46 | Osteoarthritis | 22 |
|  |  | Articular pathologies | 15 |
|  |  | Orthopaedic surgery | 6 |
|  |  | Musculoskeletal disorders | 3 |
| Dentistry and Stomatology | 17 | Feline chronic gingivostomatitis | 14 |
|  |  | Periodontal disease | 1 |
|  |  | No response | 2 |
| Neurology | 13 | Spinal cord injury | 4 |
|  |  | Intervertebral disc disease | 3 |
|  |  | Neurodegenerative disorders | 2 |
|  |  | Degenerative myelopathies | 1 |
|  |  | Cognitive syndrome | 1 |
|  |  | No response | 2 |
| Regenerative Medicine | 11 | Skin would healing | 11 |
| Oncology | 11 | Leukaemia | 2 |
|  |  | Squamous cell carcinoma | 1 |
|  |  | Canine lymphoma | 1 |
|  |  | No response | 7 |
| Nephrology | 8 | Chronic kidney disease | 7 |
|  |  | No response | 1 |
| Haematology | 7 | Bone marrow aplasia | 4 |
|  |  | anaemia | 2 |
|  |  | No response | 1 |
| Dermatology | 6 | Atopic dermatitis | 4 |
|  |  | Otitis | 1 |
|  |  | No response | 1 |
| Gastroenterology | 6 | Inflammatory bowel disease | 6 |
| Infectious diseases | 5 | Distemper | 3 |
|  |  | Leishmaniosis | 1 |
|  |  | Feline leukaemia virus | 1 |
| Immunology | 3 | Immune-mediated diseases | 3 |
| Endocrinology | 3 | Diabetes mellitus | 3 |
| Pneumology | 1 | Asthma | 1 |
| Ophthalmology | 1 | Keratoconjunctivitis sicca | 1 |

Summary of the therapeutic applications of stem cells already considered (question 22) by clinical area and specific medical conditions from 61 respondents.
